# The emerging role of apolipoprotein C-III: beyond effects on triglyceride metabolism

**DOI:** 10.1186/s12944-016-0352-y

**Published:** 2016-10-22

**Authors:** Mengdie Luo, Daoquan Peng

**Affiliations:** Department of Cardiovascular Medicine, The Second Xiangya Hospital, Central South University, Changsha, Hunan 410011 China

**Keywords:** Apolipoproteins, Atherosclerosis, Gene therapy

## Abstract

Apolipoprotein C-III has been referred to as an important participant in the metabolism of triglyceride-rich lipoproteins, leading to hypertriglyceridemia and thereafter cardiovascular disease. Accumulating evidence indicates that apolipoprotein C-III is a multifaceted protein which not only regulates triglyceride metabolism, but also participates in the atherosclerotic lesion formation and several other pathological processes involved in atherosclerosis. Based on data from experiments and clinical trials, some novel therapies such as antisense technology emerge.

## Background

Triglyceride (TG) has been identified as an important risk factor for cardiovascular disease (CVD) for a long time, but randomized controlled trials of fenofibrate and omega-3 fatty acids to reduce TG failed to show any significant clinical benefits [1, 2]. Although genome-wide association study (GWAS) has positioned some single nucleotide polymorphisms (SNPs) associated with TG to be linked with CVD, these SNPs have pleiotropic relationships with other lipids [3] and therefore TG-related effects may be confounded. The longstanding controversy renders TG not to be a persuasive biomarker in CVD pathogenesis. Renewed interest in TG is brought by recent genetic observations that loss-of-function (LOF) mutations in the gene encoding apolipoprotein C-III (apoCIII) and relevant hypotriglyceremia are associated with decreased CVD risk (40–41 %) [4, 5]. ApoCIII, an apolipoprotein composed of 79 amino acids with a molecular weight of 8.8 kDa, resides on circulating HDL, low density lipoprotein (LDL) and triglyceride-rich lipoproteins (TRLs) such as chylomicrons (CM) and very low density lipoprotein (VLDL) [6]. ApoCIII promotes hypertriglyceridemia (HTG) via different mechanisms. First, apoCIII inhibits the activity of lipoprotein lipase (LPL) and disturbs lipids lipolysis [6]. Second, apoCIII interferes with the binding of apolipoprotein B (apoB) or apolipoprotein E (apoE) to hepatic receptors, thus leading to a delayed catabolism of TRL remnants [6]. Third, apoCIII favors the assembly and secretion of VLDL in the liver [7, 8]. ApoCIII and the lipoproteins that carry apoCIII acts as not only a key regulator in the TG metabolism, but also an independent predictor for CVD risk [9, 10]. The negative effects of apoCIII agree with gene analysis in which null mutation or missense mutation of apoCIII produces apoCIII deficiency and confers lifelong cardioprotection [4, 5, 11, 12]. These evidence supports the hypothesis that apoCIII may be instrumental to mediate TG-related harmful effects. This review puts emphases on the newly-discovered function of apoCIII beyond the well-known regulator of triglyceride metabolism. The novel method to inhibit apoCIII via antisense oligonucleotide (ASO) treatment and selective capture of apoCIII via zeolite nanoparticles will be mentioned too.

## ApoCIII is directly involved in atherogenesis

### Evidence from basic experiments

#### ApoCIII facilitates interaction of monocytes and endothelial cells

**Fig. 1:**
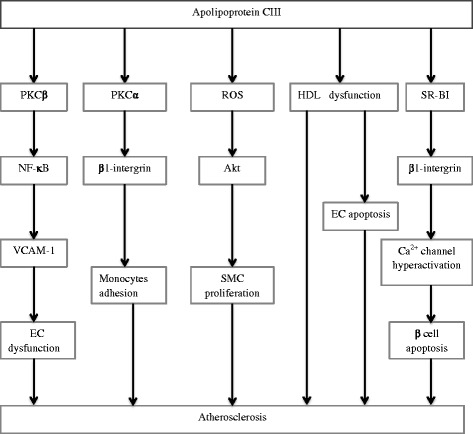
Pleiotropic effects of apoCIII. PKC indicates protein kinase C; NF-κB, nuclear factor-κB; VCAM-1, vascular cell adhesion molecule-1; EC, endothelial cells; ROS, reactive oxygen species; SMC, smooth muscle cells; HDL, high density lipoprotein; SR-BI, scavenger receptor class B type I

Interaction between monocytes and endothelial cells (ECs) is the initial step in the formation of atherosclerotic lesion [13]. ApoCIII itself and apoB lipoproteins containing apoCIII could activate pertussis toxin (PTX)-sensitive G protein pathway to activate phosphatidylcholine-specific phospholipase (PC-PLC). PC-PLC can catalyze PC to phospholylcholine and diacylglycerol (DAG) , and then DAG induces protein kinase C (PKC) α activation and leads to activation of nuclear factor-κB (NF-κB) [14], which increases β1-integrin expression in monocytes and enhances their adhesion to ECs. Besides, apoCIII also activates PKC β, leading to NF-κB activation and vascular cell adhesion molecule-1 (VCAM-1) expression in ECs, which contributes to EC dysfunction and monocyte recruitment [15] (Fig. 1). All of these findings can be demonstrated not only under static conditions but also under laminar flow. Since apoCIII can directly affect the interaction between monocytes and ECs, more attention is given to the direct atherogenic effects of apoCIII. A recent study by Zheng et al. extends the experiments from venous endothelium to coronary artery endothelium and provides in vivo evidence for the independent atherogenic effects of apoCIII and atheroprotective effects of statins because of decreasing apoCIII [16]. The study has demonstrated that apoCIII can promote NF-κB and subsequent VCAM-1 expression, leading to EC activation and monocytes adhesion, all of which can be abolished by statin effectively [16]. Although these findings seem to have built a complete line of evidence to explain the effects of apoCIII on vascular inflammation, many questions, for example, how apoCIII activates PTX-sensitive G protein , how apoCIII activates PKC β, are there any other mediators used by apoCIII to activate PKC α beyond PTX senstive G protein, are there any other molecules can also be affected by statins to reduce vascular inflammation, still require further exploration.

#### ApoCIII promotes smooth muscle cell proliferation

Smooth muscle cells (SMCs) have been identified as an crucial participant in the progression of atherosclerotic lesion and neointimal formation. A recently published article demonstrates that apoCIII can promote SMC proliferation via Akt signalling pathway mediated by reactive oxygen species in vitro, leading to aggravated restenosis and atherogenesis [17] (Fig. 1). The researchers built reliable HTG models through human apoCIII transgenic (apoCIIItg) mice and observed significant atherosclerotic lesion development in apoCIIItg mice with LDL receptor deficient background. Furthermore, they crossed apoCIIItg or apoCIII−/−mice with glycosylphosphatidylinositol-anchored high density lipoprotein-binding protein 1-deficient (GPIHBP1−/−) mice, which exhibited extreme HTG because of impaired LPL anchoring to ECs, and harvested authentic TRL with or without apoCIII (TRL+ apoCIII or TRL–apoCIII) from the crossed mice to further distinguish the effect between TRL and apoCIII without impairing TRL properties. It was shown that TRL+ apoCIII promoted SMC proliferation while TRL–apoCIII not. The study adds more weight to the hypothesis that apoCIII can aggravate atherogenesis directly. However, no significant difference exists between apoCIII−/−and WT mice, which seems contradicted with findings from human researches that apoCIII LOF mutation provides cardioprotection [4, 5]. This may be caused by species difference that deletion of mouse apoCIII is not the same as apoCIII LOF mutation in human. Overall, more experiments are still needed to address the role of human apoCIII on human atherogenesis and restenosis.

### Evidence from clinical studies

#### ApoCIII alters platelet activity

Platelets and the coagulation system are importantly involved with atherogenesis and atherothrombosis [18]. Results from a prospective study concerning 663 subjects with angiographically proven CVD suggest that basal serum concentration of apoCIII ≥ 10.5 mg/dl represents an independent risk factor of overall and cardiovascular mortality in a 5-year follow-up [19] (Table 1). Besides, the sub-analysis from this study reveals an independent association between elevated apoCIII and enhanced thrombin generation [19]. Moreover, another retrospective study about 933 unrelated patients enrolled in Verona heart Study has found that increased concentration of apoCIII is associated with elevated thrombin level in the plasma even after adjustment for other risk factors and apoCIII presents similar correlation with the presence of Factor II (FII) G20210A gene variant, the most important genetic determinant of FII and hereditary risk factor for venous thrombosis [20] (Table 1). It’s noteworthy that no significant relationship has been observed between thrombin activity and TRLs [20]. Although no causal mechanism could be inferred from these analysis because of the intrinsically flaw of cross-sectional design, they indicate that apoCIII could alter the homeostatic balance in a procoagulant way and may promote atherothrombotic complications.

**Table 1:** Summary of studies investigating the relationship between apoCIII and cardiovascular risk

Study	Study design	Study population (n)	Mean age (y)	Male (%)	apoCIII levels (mg/dl)		Lipid levels (mg/dl)				Key study findings
					In plasma	In HDL	TG	TC	HDL-C	LDL-C	
Olivieri et al. [19]	prospective study	CVD patients (*n* = 633)	60.2	81.5 %	11.3^a^	NR	154.9	220.8	46.3	147.9	Serum apoCIII ≥ 10.5 mg/dl represents an independent risk factor of overall (HR, 2.22; 95 % CI, 1.16–4.24; *P* = 0.016) and cardiovascular mortality (HR, 2.35; 95 % CI, 1.19–4.62; *P* = 0.014).
Olivieri et al. [20]	retrospective study	CVD patients (*n* = 687) vs CVD-free patients (*n* = 246)	60.6 vs 57.5	81.0 % vs 71.0 %	11.4 vs 10.2	NR	152.2 vs 117.7	221.2 vs 212.7	46.7 vs 55.6	147.9 vs 137.1	Increased concentration of apoCIII is associated with elevated risk of venous thrombosis (β, 0.192; 95 % CI, 0.109–0.274; *P* < 0.001).
Jensen et al. [22]	case-control study	CVD patients (NHS-women: *n* = 286; HPFS-men: *n* = 348) vs controls (NHS-women: *n* = 286; HPFS-men: *n* = 351)	NHS-women: 60.2 vs 60.1; HPFS-men: 64.3 vs 64.2	54.9 % vs 55.1 %	NR	NHS-women: 12 vs 12; HPFS-men: 12 vs 11	NHS-women: 106.2 vs 88.5; HPFS-men: 115.0 vs 97.3	NHS-women: 234.4 vs 230.5; HPFS-men: 218.1 vs 209.3	NHS-women: 68.0 vs 72.6; HPFS-men: 46.7 vs 48.6	NHS-women: 148.3 vs 142.5; HPFS-men: 135.1 vs 125.9	HDL with apoCIII (OR, 1.18; 95 % CI, 1.03–1.34; *P* = 0.01) and HDL without apoCIII (OR, 0.66; 95 % CI, 0.53–0.83; *P* = 0.0001) are oppositely related to CVD risk.
Talayero et al. [23]	case-control study	Obese subjects (*n* = 20) vs normal weight subjects (*n* = 20)	51 vs 51	35 % vs 35 %	13.2 vs 7.5	11.9 vs 6.7	153 vs 83	210 vs 187	50 vs 65	130 vs 105	ApoCIII containing HDL particles (11.4 vs 5.2) and apoCIII concentration in HDL particles (11.9 vs 6.7) are significantly higher in obese subjects.
Chang et al. [24]	case-control study	CVD patients (*n* = 90) vs non-CVD patients (*n* = 200)	57.4 vs 53.4	80 % vs 48 %	17.4 vs 18.9	12.3 vs 9.4	261.9 vs 223.2	244.8 vs 224.3	37.0 vs 42.5	174.6 vs 165.5	HDL apoCIII to VLDL apoCIII ratio is considered as a more reliable marker to predict CVD (OR, 2.04; 95 % CI, 1.46–2.84; *P* < 0.0001).
Xiong et al. [25]	case-control study	CVD patients (*n* = 120) vs Non-CVD patients (*n* = 80)	55.5 vs 51.3	76.7 % vs 55.0 %	12.0 vs 12.7	25.1vs 21.0^b^	155.8 vs 143.4	173.4 vs 181.9	39.4 vs 47.9	111.2 vs 117.0	HDL apoCIII is an independent predictor of CVD (OR, 1.04; 95 % CI, 1.00–1.08; *P* = 0.039).

*ApoCIII* indicates apolipoprotein CIII, *HDL* high density lipoprotein, *TG* triglyceride, *TC* total cholesterol, *LDL-C* low density lipoprotein-cholesterol, *CVD* cardiovascular disease, *NHS* Nurses’ Health Study, *HPFS* Health Professional Follow-up Study, *NR* not reported

^a^in serum

^b^ug/mg HDL

## ApoCIII modifies risk factors of atherosclerosis

### Evidence from clinical studies

#### HDL dysfunction

HDL has been reported to exert various atheroprotective properties, including anti-apoptotic effects, anti-inflammatory effects and cholesterol efflux capacity [21]. All of these can be altered by pathological disorders and HDL with defects in these properties is referred to as dysfunctional HDL, which in turn contributes to the disorders, especially CVD.

Accumulating evidence suggests that apoCIII correlates tightly with HDL dysfunction. A prospective case-control study has identified two types of HDL: HDL with apoCIII and HDL without apoCIII, and the two HDL subgroups are oppositely related to CVD risk. The adjusted relative risk per standard deviation for HDL-C with apoCIII is 1.18 (1.03–1.34) and for HDL-C without apoCIII is 0.66 (0.53–0.83) [22] (Table 1). In a comparative study, apolipoprotein A-I containing HDL particles with apoCIII is significantly higher in obese subjects than normal weight (10 % vs. 4 %, *P* = 0.009) and apoCIII concentration in these HDL particles is 1.5–2 times higher in obese subjects (*P* ≤ 0.004), both of which indicated increased risk for CVD [23] (Table 1). In the Chin-Shan Community Cardiovascular Cohort study in Taiwan, HDL apoCIII to VLDL apoCIII ratio is considered as a more reliable marker to predict CVD than conventional apolipoproteins or lipid factors (odds ratio: 2.04; 95 % CI: 1.46–2.84; *P* < 0.0001) [24] (Table 1).

Besides, the concentration of apoCIII in HDL is significantly higher in CVD patients than in non-CVD patients according to the results from enzyme-linked immunosorbent assay method [25] (Table 1) and mass spectrometry [26]. ApoCIII increase in HDL from CVD patients can activate mitogen-activated protein kinase (MAPK) signalling pathway via phosphorylation of p38 in ECs and thus increase the expression of pro-apoptotic protein tBID, altering HDL from anti-apoptotic to pro-apoptotic [26] (Fig. 1).

The HDL dysfunction related to apoCIII is not only seen in CVD patients, but also seen in patients with chronic kidney disease (CKD). Holzer et al. found that apoCIII was highly abundant in HDL isolated from hemodialysis patients and correlated inversely with HDL cholesterol efflux capacity [27]. However, the study did not analyze the relationship between apoCIII and other changed HDL components which may also affect HDL cholesterol efflux capacity in CKD. Therefore, it’s unreliable to justify that enrichment of HDL with apoCIII would impair HDL cholesterol efflux capacity independent of other HDL components.

These findings indicate that apoCIII increases in HDL may exert some disruptive impacts on HDL functional properties and provide a novel mechanism for HDL dysfunction.

### Evidence from basic experiments

#### Diabetes mellitus

Patients with diabetes mellitus often display abnormal lipid profiles and are susceptible to CVD. Elevated glucose can upregulate apoCIII expression in the transcriptional level via the activation of transcription factors carbohydrate response element-binding protein and hepatocyte nuclear factor-4α and thus plasma apoCIII concentration presents a positive correlation with fasting plasma glucose as well as glucose excursion after oral glucose load in obese humans [28]. However, the relationship between insulin and apoCIII still remains controversial.

In vitro experiments have shown that apoCIII interacted with scavenger-receptor BI (SR-BI) and then upregulated β1 integrin expression, leading to the hyperactivation of β cell Ca^2+^ channels through the coactivation of PKA and Src kinase pathways [29] (Fig. 1). This finding has been supported by another study carried on a special animal model diabetes-prone BB rat, which develops type 1 diabetes mellitus (T1DM) at around the age of 60 days spontaneously [30]. Of note, antisense treatment to decrease the level of endogenous apoCIII can delay the onset of T1DM [30], which suggests that apoCIII is an essential participant in the development of T1DM.

As for T2DM, it’s well-known that it is featured by two major defects: insulin resistance and β cell failure. A cross-sectional study about 1422 T2DM subjects found that apoCIII correlated positively with coronary artery calcification, a biomarker of subclinical atherosclerosis [31]. Juntti-Berggren et al. found that islet insulin resistance could promote local apoCIII production [32]. Besides, they transplanted apoCIII knockout islet and islet from ob/ob or B6 mice into the anterior chamber of the eye in an ob/ob mouse or a HFD-treated B6 mice, both of which were characterized by high systemic serum levels of apoCIII and impaired glucose tolerance [32]. By this way a delicate system was created where the islets rich and deficient in apoCIII coexisted in one animal model . The elevation of intraislet apoCIII production increased local inflammation, deranged Ca^2+^ handling in β cells and thus β cell apoptosis [32]. From this perspective, ApoCIII serves as a bridge linking insulin resistance and β cell failure in T2DM. Besides, systemically decreasing apoCIII in vivo via ASO treatment can improve glucose tolerance in T2DM [32].

In contrast to the studies above, one study investigated effects of apoCIII on intact rat pancreatic islets in the presence of proinflammatory cytokines ( interferon-γ + interleukin 1β) to find that apoCIII reduced β cell apoptosis by stimulating phosphorylation of Akt [33]. The disagreement may be caused by the differences about experiment factors such as models and intervention methods. Of note, it’s inappropriate to draw a conclusion from one study that apoCIII may exert some protective function on islet cells.

#### Lp-PLA2

Although whether lipoprotein-associated phospholipase A2(Lp-PLA2) is an independent risk factor for atherosclerosis remains controversial, Lp-PLA2 activity and mass shows a strong, positive association with atherosclerosis [34]. Results from in vitro experiments and animal models revealed that apoCIII could upregulate Lp-PLA2 expression via MAPK and NF-kB pathways in a dose- and time-dependent manner and thus induce inflammation [35]. In addition, in vitro experiments indicated that the downstream Lp-PLA2 could also increase apoCIII expression in the liver in turn [35]. However, the detailed mechanisms in the circle still await further investigation.

## Future prospects

Species-specific antisense suppression of apoCIII has produced robust reduction in serum apoCIII and TG and demonstrated good tolerance in various preclinical models, including human apoCIII transgenic mice, rats, mouse, nonhuman primates [36]. In the phase I clinical study in healthy human volunteers, administration of human apoCIII ASO (volanesorsen, formally ISIS-APOCIIIRx) brought about a profound dose- and time-dependent reduction of apoCIII and TG without remarkable adverse effects [36]. The phase 2 study evaluated the pharmacodynamic effects of volanesorsen in adult patients with severe or uncontrolled HTG [37]. When volanesorsen was administrated as a single agent, plasma apoCIII was reduced on apoB100 and apoAI containing lipoproteins as well as lipoprotein (a) uniformly [38] in a dose-dependent fashion (apoCIII: 40 %-100 mg, 63.8 %-200 mg, 79.6 %-300 mg). TG decreased in concordance with apoCIII (TG: 31.3 %-100 mg, 57.7 %-200 mg, 70.9 %-300 mg) and showed a strong, positive correlation with apoCIII, while HDL-C presented a dose-dependent increase (HDL-C: 36.6 %-100 mg, 36.2 %-200 mg, 45.7 %-300 mg). Similar changes occurred when volanesorsen was given as an add-on to stable fibrate therapy. No serious treatment-associated safety issues were identified. The apparent clinical benefits brought by volanesorsen presented not only in patients with HTG but also in those with familial chylomicronemia syndrome (FCS). In FCS patients, absence of LPL caused insufficient TRLs removal and resulted in chylomicronemia (TG > 880 mg/dl). ASO treatment exhibited substantial efficacy to lower TG less than 500 mg/dl independent of LPL [39]. Short-term efficacy and apparent safety of volanesorsen brings light to further long-term evaluation. Ongoing phase III trials about volanesorsen include the APPROACH (the APPROACH study: A Study of ISIS-APOCIIIRx in Patients with Famial Chylomicronemia Syndrome, NCT02211209), the COMPASS (the COMPASS study: A Study of Volanesorsen in Patietns with Hypertriglyceridemia, NCT02300233), and the BROADEN (the BROADEN study: A Study of Volanesorsen in Patients with Partial Lipodystrophy, NCT02527343). These studies aim to evaluate the efficacy and safety of 300 mg volanesorsen versus placebo administered subcutaneously once a week for 52 ,26, 52 weeks respectively in patients with corresponding diseases. The primary outcome is measured by the percent change in fasting TG from baseline to 13 weeks. ApoCIII ASO treatment links genetic insights to molecular mechanisms and keeps abreast with the ‘precision medicine’.

In addition to the promising ASO treatment, zeolite nanoparticles, whose negative charge help it to interact electrostatically with the positively charged amino acid residues of apoCIII , selectively capture plasma apoCIII and may be a potential therapy to reduce plasma apoCIII in the future [40].

## Conclusions

Considerable evidence indicates that apoCIII can affect the development of CVD not only by regulating triglyceride metabolism but also through its direct atherogenic effects. Novel understanding of apoCIII in atherogenesis makes it an attractive therapy target and some innovative approaches to modifying it emerge. Although full illustration of apoCIII function still awaits further exploration, to lower the apoCIII levels in circulation via specific intervention seems to be an intriguing therapy for cardiovascular protection.

## Abbreviations

apoB: apolipoprotein B
apoCIII: apolipoprotein C-III
apoE: apolipoprotein E
ASO: Antisense oligonucleotide
CKD: Chronic kidney disease
CM: Chylomicron
CVD: Cardiovascular disease
DAG: Diacylglycerol
EC: Endothelial cell
FCS: Familial chylomicronemia syndrome
GPIHBP1: Glycosylphosphatidylinositol-anchored high density lipoprotein-binding protein 1
GWAS: Genome-wide association study
HTG: Hypertriglyceridemia
LDL: Low density lipoprotein
LOF: Loss-of-function
LPL: Lipoprotein lipase
Lp-PLA2: Lipoprotein-associated phospholipase A2
NF-κB: Nuclear factor-κB
PC-PLC: Phosphatidylcholine-specific phospholipase
PKC: Protein kinase C
PTX: Pertussis toxin
SMC: Smooth muscle cell
SNP: Single nucleotide polymorphism
SR-BI: Scavenger-receptor BI
T1DM: Type 1 diabetes mellitus
TG: Triglyceride
TRL: Triglyceride-rich lipoproteins
VCAM-1: Vascular cell adhesion molecule-1
VLDL: Very low density lipoprotein
